# 1,2,3,4-Tetra­hydro­isoquinoline-2-sulfonamide

**DOI:** 10.1107/S1600536807068158

**Published:** 2008-01-11

**Authors:** Radia Bouasla, Malika Berredjem, Nour-Eddine Aouf, Carole Barbey

**Affiliations:** a Laboratoire de Chimie Organique Appliquée, LCOA, Groupe de Chimie Bioorganique, Faculté des Sciences, Département de Chimie, Université d’Annaba, Algeria; b Laboratoire de Biophysique Moléculaire Cellulaire et Tissulaire (UMR 7033 CNRS), UFR-SMBH Université Paris-Nord, 74 rue M. Cachin, 93017 Bobigny Cedex, France

## Abstract

The title compound, C_9_H_12_N_2_O_2_S, is a useful precursor of a variety of modified sulfonamide mol­ecules. Due to the importance of these mol­ecules in biological systems (antibacterials, antidepressants and many other applications), there is a growing inter­est in the discovery of new biologically active compounds. In the title compound, the mol­ecules are linked by N—H⋯O inter­molecular hydrogen bonds involving the sulfonamide function to form an infinite two-dimensional network parallel to the (001) plane.

## Related literature

For related literature, see: Berredjem *et al.* (2000 ▶); Lee & Lee (2002 ▶); Martinez *et al.* (2000 ▶); Xiao & Timberlake (2000 ▶); Esteve & Bidal (2002 ▶); Soledade *et al.* (2006 ▶).
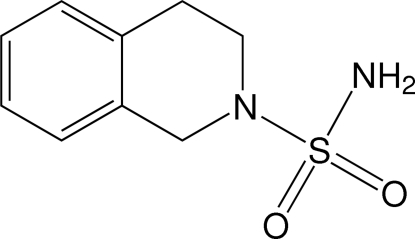


## Experimental

### 

#### Crystal data

C_9_H_12_N_2_O_2_S
*M* *_r_* = 212.27
Monoclinic, 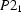

*a* = 5.275 (1) Å
*b* = 9.541 (1) Å
*c* = 10.229 (1) Å
β = 101.80 (5)°
*V* = 503.93 (15) Å^3^
*Z* = 2
Mo *K*α radiation
μ = 0.30 mm^−1^
*T* = 293 (2) K
0.10 × 0.10 × 0.10 mm

#### Data collection

Nonius KappaCCD diffractometer
Absorption correction: none
8285 measured reflections
2210 independent reflections
2106 reflections with *I* > 2σ(*I*)
*R*_int_ = 0.032

#### Refinement

*R*[*F* ^2^ > 2σ(*F* ^2^)] = 0.030
*wR*(*F* ^2^) = 0.087
*S* = 1.13
2210 reflections
127 parameters
1 restraint
H-atom parameters constrained
Δρ_max_ = 0.25 e Å^−3^
Δρ_min_ = −0.30 e Å^−3^
Absolute structure: Flack (1983 ▶), 979 Friedel pairs
Flack parameter: −0.01 (6)

### 

Data collection: *COLLECT* (Hooft, 1998 ▶); cell refinement: *DENZO*/*SCALEPACK* (Otwinowski & Minor, 1997 ▶); data reduction: *COLLECT*; program(s) used to solve structure: *SHELXS97* (Sheldrick, 1997 ▶); program(s) used to refine structure: *SHELXL97* (Sheldrick, 1997 ▶); molecular graphics: *ORTEP-3 for Windows* (Farrugia, 1997 ▶) and *PLATON* (Spek, 2003 ▶); software used to prepare material for publication: *SHELXL97* and *CrystalBuilder* (DECOMET Laboratory, 2007 ▶).

## Supplementary Material

Crystal structure: contains datablocks global, I. DOI: 10.1107/S1600536807068158/dn2304sup1.cif

Structure factors: contains datablocks I. DOI: 10.1107/S1600536807068158/dn2304Isup2.hkl

Additional supplementary materials:  crystallographic information; 3D view; checkCIF report

## Figures and Tables

**Table 1:** Hydrogen-bond geometry (Å, °)

*D*—H⋯*A*	*D*—H	H⋯*A*	*D*⋯*A*	*D*—H⋯*A*
N2—H21⋯O1^i^	0.91	2.03	2.928 (2)	173
N2—H22⋯O2^ii^	0.92	2.10	2.971 (2)	159

Symmetry codes: (i) 
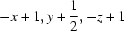
; (ii) 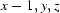.

**Fig. 1.**
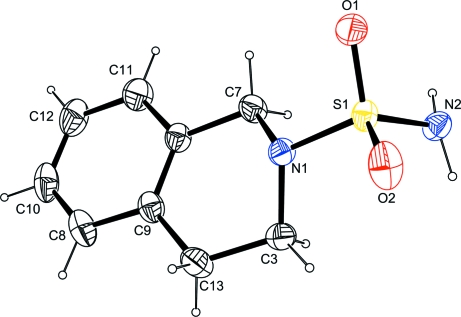
Molecular View of the title compound. Displacement ellipsoids are drawn at the 30% probability level. H atoms are represented as small spheres of arbitrary radii.

**Fig. 2.**
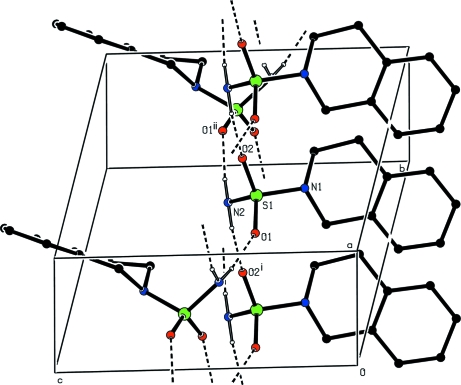
Partial packing view showing the formation of the two dimensional network. H bonds are represented as dashed lines. H atoms not involved in hydrogen bonding have been omitted for clarity. [Symmetry codes: (i) x - 1, y, z; (ii) -x + 1, y + 1/2, -z + 1]

**Fig. 3.**
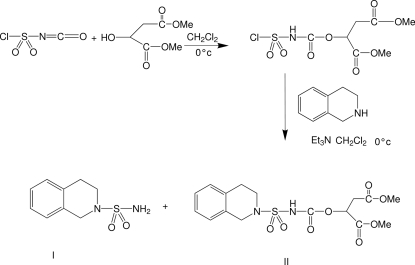
Chemical pathway of the formation of (I)

## supplementary crystallographic information

### Comment

The sulfamide unit is an ubiquitous structural entity in many naturally occurring compounds and medicinal agents (*i.e.* anticonvulsant, antihypertensive, hypoglycemic agents, histamine H2-receptor antagonist, herbicide, human cytomegalovirus inibitors···) (Soledade *et al.*, 2006; Esteve & Bidal, 2002; Xiao & Timberlake, 2000; Martinez *et al.*, 2000; Berredjem *et al.*, 2000; Lee *et al.*, 2002) We report herein the synthesis and the crystal structure determination of the title compound (Fig. 1).

The crystal structure consists of layers of hydrophobic regions that enclose the bicyclic moiety and polar regions where the sulfamide atoms are involved in hydrogen bond network. Namely, the sulfamide group is involved in four hydrogen bonds (2 with sulfamide O atoms, 2 with nitrogen atom) with four different symmetry-related molecules, building a two dimensional network parallel to the (0 0 1) plane (Table 1, Fig. 2).

### Experimental

A solution of dimethyl malate (2,27 g, 14.1 mmol) in anhydrous CH_2_Cl_2_ (10 ml) was added to a stirring solution of chlorosulfonyl isocyanate (1.23 ml, 14.1 mmol) in CH_2_Cl_2_ (10 ml) at 0°C dropwise over period of 10 min. The resulting solution was transferred to a mixture of 1, 2, 3, 4 tetrahydroquinoleine (1,87 g, 14,1 mmol) in CH_2_Cl_2_ (20 ml) in the presence of triethylamine (1.1 equiv.). The solution was stirred at 0°C for less than 1.5 h. The reaction mixture was washed with HCl 0.1 N and water, and the organic layer was dried over anhydrous sodium sulfate, filtered and concentrated *in vacuo*. Two compounds were obtained after purification by silica gel chromatography (Fig. 3). Slow evaporation at room temperature of a concentrated dichloromethane / methanol (9/1) solution of the most polar product (sulfamide I) afforded yellow crystals suitable for diffraction.

### Refinement

All H atoms attached to C atoms were fixed geometrically and treated as riding with C—H = 0.93 Å (aromatic) or 0.97 Å (methylene) with *U*_iso_(H) = 1.2*U*_eq_(C). H atoms of amino group were located in difference Fourier maps and included in the subsequent refinement using restraints (N—H= 0.90 (1)Å and H···H= 1.66 (2) Å) with *U*_iso_(H) = 1.2*U*_eq_(N). In the last stage of refinement, they were treated as riding on their parent N atom.

### Crystal data

**Table:**

C_9_H_12_N_2_O_2_S	*F*_000_ = 224
*M**_r_* = 212.27	*D*_x_ = 1.399 Mg m^−^^3^
Monoclinic, *P*2_1_	Mo *K*α radiation λ = 0.71070 Å
Hall symbol: P 2yb	Cell parameters from 6025 reflections
*a* = 5.275 (1) Å	θ = 2.0–27.5º
*b* = 9.541 (1) Å	µ = 0.30 mm^−^^1^
*c* = 10.229 (1) Å	*T* = 293 (2) K
β = 101.80 (5)º	Parallelepipedic, yellow
*V* = 503.93 (15) Å^3^	0.10 × 0.10 × 0.10 mm
*Z* = 2	

### Data collection

**Table:**

Nonius KappaCCD diffractometer	2210 independent reflections
Radiation source: fine-focus sealed tube	2106 reflections with *I* > 2σ(*I*)
Monochromator: graphite	*R*_int_ = 0.032
Detector resolution: 9 pixels mm^-1^	θ_max_ = 27.5º
*T* = 293(2) K	θ_min_ = 2.0º
φ and ω scans	*h* = −6→6
Absorption correction: none	*k* = −12→11
8285 measured reflections	*l* = −13→13

### Refinement

**Table:**

Refinement on *F*^2^	Hydrogen site location: inferred from neighbouring sites
Least-squares matrix: full	H-atom parameters constrained
*R*[*F*^2^ > 2σ(*F*^2^)] = 0.030	*w* = 1/[σ^2^(*F*_o_^2^) + (0.0575*P*)^2^ + 0.0148*P*] where *P* = (*F*_o_^2^ + 2*F*_c_^2^)/3
*wR*(*F*^2^) = 0.087	(Δ/σ)_max_ < 0.001
*S* = 1.13	Δρ_max_ = 0.25 e Å^−^^3^
2210 reflections	Δρ_min_ = −0.30 e Å^−^^3^
127 parameters	Extinction correction: none
1 restraint	Absolute structure: Flack (1983), 979 Friedel pairs
Primary atom site location: structure-invariant direct methods	Flack parameter: −0.01 (6)
Secondary atom site location: difference Fourier map	

### Special details

**Table:**

Geometry. All e.s.d.'s (except the e.s.d. in the dihedral angle between two l.s. planes) are estimated using the full covariance matrix. The cell e.s.d.'s are taken into account individually in the estimation of e.s.d.'s in distances, angles and torsion angles; correlations between e.s.d.'s in cell parameters are only used when they are defined by crystal symmetry. An approximate (isotropic) treatment of cell e.s.d.'s is used for estimating e.s.d.'s involving l.s. planes.
Refinement. Refinement of *F*^2^ against ALL reflections. The weighted *R*-factor *wR* and goodness of fit *S* are based on *F*^2^, conventional *R*-factors *R* are based on *F*, with *F* set to zero for negative *F*^2^. The threshold expression of *F*^2^ > σ(*F*^2^) is used only for calculating *R*-factors(gt) *etc*. and is not relevant to the choice of reflections for refinement. *R*-factors based on *F*^2^ are statistically about twice as large as those based on *F*, and *R*- factors based on ALL data will be even larger.

### Fractional atomic coordinates and isotropic or equivalent isotropic displacement parameters (Å^2^)

**Table:**

	*x*	*y*	*z*	*U*_iso_*/*U*_eq_	
S1	0.56649 (7)	0.52966 (4)	0.42439 (3)	0.03710 (13)	
N1	0.4887 (3)	0.60389 (14)	0.27757 (14)	0.0369 (3)	
C3	0.5408 (4)	0.7557 (2)	0.2719 (2)	0.0475 (5)	
H3A	0.6993	0.7792	0.3345	0.057*	
H3B	0.3998	0.8087	0.2956	0.057*	
O2	0.8298 (3)	0.5665 (2)	0.47635 (14)	0.0624 (5)	
C6	0.1947 (4)	0.6250 (2)	0.06005 (17)	0.0415 (4)	
C7	0.2282 (4)	0.5698 (2)	0.20079 (17)	0.0451 (4)	
H7A	0.0985	0.6112	0.2439	0.054*	
H7B	0.2038	0.4690	0.1987	0.054*	
C8	0.3113 (5)	0.7727 (3)	−0.1068 (2)	0.0608 (6)	
H8	0.4195	0.8406	−0.1311	0.073*	
C9	0.3522 (4)	0.7280 (2)	0.02630 (18)	0.0442 (4)	
C10	0.1146 (5)	0.7182 (3)	−0.2021 (2)	0.0631 (6)	
H10	0.0888	0.7499	−0.2898	0.076*	
C11	−0.0028 (5)	0.5703 (3)	−0.0374 (2)	0.0611 (6)	
H11	−0.1098	0.5009	−0.0146	0.073*	
C12	−0.0432 (5)	0.6172 (3)	−0.1677 (2)	0.0669 (7)	
H12	−0.1773	0.5802	−0.2317	0.080*	
C13	0.5667 (4)	0.7918 (2)	0.1305 (2)	0.0538 (5)	
H13A	0.5636	0.8929	0.1200	0.065*	
H13B	0.7325	0.7582	0.1162	0.065*	
O1	0.4929 (3)	0.38595 (15)	0.40334 (14)	0.0580 (4)	
N2	0.4032 (3)	0.59065 (17)	0.52808 (16)	0.0426 (3)	
H21	0.4478	0.6796	0.5540	0.051*	
H22	0.2355	0.5596	0.5109	0.051*	

### Atomic displacement parameters (Å^2^)

**Table:**

	*U*^11^	*U*^22^	*U*^33^	*U*^12^	*U*^13^	*U*^23^
S1	0.0370 (2)	0.0412 (2)	0.03170 (18)	0.00882 (17)	0.00373 (13)	0.00083 (17)
N1	0.0378 (8)	0.0365 (8)	0.0340 (6)	0.0022 (6)	0.0012 (5)	0.0019 (6)
C3	0.0564 (12)	0.0391 (10)	0.0451 (10)	−0.0060 (8)	0.0055 (9)	0.0004 (8)
O2	0.0313 (7)	0.1054 (14)	0.0475 (7)	0.0094 (7)	0.0011 (5)	0.0075 (8)
C6	0.0424 (10)	0.0444 (9)	0.0356 (8)	0.0066 (8)	0.0029 (7)	0.0031 (7)
C7	0.0430 (9)	0.0500 (11)	0.0385 (8)	−0.0071 (8)	−0.0008 (7)	0.0080 (7)
C8	0.0667 (15)	0.0737 (15)	0.0458 (11)	0.0099 (12)	0.0204 (11)	0.0167 (11)
C9	0.0457 (9)	0.0485 (10)	0.0399 (8)	0.0096 (8)	0.0122 (8)	0.0069 (8)
C10	0.0767 (15)	0.0790 (15)	0.0340 (9)	0.0256 (13)	0.0121 (10)	0.0077 (10)
C11	0.0631 (13)	0.0690 (14)	0.0434 (10)	−0.0081 (11)	−0.0069 (9)	0.0031 (9)
C12	0.0745 (15)	0.0789 (17)	0.0395 (10)	0.0129 (13)	−0.0066 (10)	−0.0040 (10)
C13	0.0525 (12)	0.0564 (13)	0.0514 (11)	−0.0089 (10)	0.0080 (9)	0.0130 (10)
O1	0.0933 (12)	0.0335 (7)	0.0448 (7)	0.0131 (7)	0.0087 (7)	0.0030 (6)
N2	0.0424 (8)	0.0439 (8)	0.0431 (8)	−0.0024 (6)	0.0122 (6)	−0.0091 (7)

### Geometric parameters (Å, °)

**Table:**

S1—O2	1.4261 (16)	C8—C10	1.373 (4)
S1—O1	1.4293 (16)	C8—C9	1.401 (3)
S1—N2	1.6060 (16)	C8—H8	0.9300
S1—N1	1.6350 (14)	C9—C13	1.515 (3)
N1—C7	1.473 (2)	C10—C12	1.366 (4)
N1—C3	1.478 (2)	C10—H10	0.9300
C3—C13	1.520 (3)	C11—C12	1.380 (3)
C3—H3A	0.9700	C11—H11	0.9300
C3—H3B	0.9700	C12—H12	0.9300
C6—C9	1.376 (3)	C13—H13A	0.9700
C6—C11	1.388 (3)	C13—H13B	0.9700
C6—C7	1.509 (2)	N2—H21	0.9059
C7—H7A	0.9700	N2—H22	0.9154
C7—H7B	0.9700		
			
O2—S1—O1	120.43 (11)	C10—C8—C9	121.3 (2)
O2—S1—N2	106.12 (10)	C10—C8—H8	119.4
O1—S1—N2	106.34 (10)	C9—C8—H8	119.4
O2—S1—N1	106.13 (10)	C6—C9—C8	118.7 (2)
O1—S1—N1	105.53 (8)	C6—C9—C13	120.86 (17)
N2—S1—N1	112.45 (9)	C8—C9—C13	120.4 (2)
C7—N1—C3	110.85 (15)	C12—C10—C8	119.7 (2)
C7—N1—S1	115.19 (12)	C12—C10—H10	120.1
C3—N1—S1	116.54 (12)	C8—C10—H10	120.1
N1—C3—C13	108.23 (16)	C12—C11—C6	121.1 (2)
N1—C3—H3A	110.1	C12—C11—H11	119.5
C13—C3—H3A	110.1	C6—C11—H11	119.5
N1—C3—H3B	110.1	C10—C12—C11	119.7 (2)
C13—C3—H3B	110.1	C10—C12—H12	120.1
H3A—C3—H3B	108.4	C11—C12—H12	120.1
C9—C6—C11	119.43 (18)	C9—C13—C3	112.27 (17)
C9—C6—C7	122.01 (17)	C9—C13—H13A	109.1
C11—C6—C7	118.57 (18)	C3—C13—H13A	109.1
N1—C7—C6	110.24 (15)	C9—C13—H13B	109.1
N1—C7—H7A	109.6	C3—C13—H13B	109.1
C6—C7—H7A	109.6	H13A—C13—H13B	107.9
N1—C7—H7B	109.6	S1—N2—H21	113.0
C6—C7—H7B	109.6	S1—N2—H22	112.6
H7A—C7—H7B	108.1	H21—N2—H22	122.9

### Hydrogen-bond geometry (Å, °)

**Table:**

*D*—H···*A*	*D*—H	H···*A*	*D*···*A*	*D*—H···*A*
N2—H21···O1^i^	0.91	2.03	2.928 (2)	173
N2—H22···O2^ii^	0.92	2.10	2.971 (2)	159

Symmetry codes: (i) −*x*+1, *y*+1/2, −*z*+1; (ii) *x*−1, *y*, *z*.
